# The ups and downs of temporal orienting: a review of auditory temporal orienting studies and a model associating the heterogeneous findings on the auditory N1 with opposite effects of attention and prediction

**DOI:** 10.3389/fnhum.2013.00263

**Published:** 2013-06-11

**Authors:** Kathrin Lange

**Affiliations:** Institut für Experimentelle Psychologie, Heinrich-Heine-Universität DüsseldorfDüsseldorf, Germany

**Keywords:** temporal orienting, attention, predictability, audition, ERP, N1

## Abstract

The temporal orienting of attention refers to the process of focusing (neural) resources on a particular time point in order to boost the processing of and the responding to sensory events. Temporal attention is manipulated by varying the task-relevance of events at different time points or by inducing expectations that an event occurs at a particular time point. Notably, the electrophysiological correlates of these manipulations at early processing stages are not identical: Auditory studies operationalizing temporal attention through task-relevance consistently found enhancements of early, sensory processing, as shown in the N1 component of the auditory event-related potential (ERP). By contrast, previous work on temporal orienting based on expectations showed mixed results: early, sensory processing was either enhanced or attenuated or not affected at all. In the present work, I will review existing findings on temporal orienting with a special focus on the auditory modality and present a working model to reconcile the previously heterogeneous results. Specifically, I will suggest that when expectations are used to manipulate attention, this will lead both to an orienting of attention and to the generation of precise predictions about the upcoming event. Attention and prediction are assumed to have opposite effects on early auditory processing, with temporal attention increasing and temporal predictions decreasing the associated ERP correlate, the auditory N1. The heterogeneous findings of studies manipulating temporal orienting by inducing expectations may thus be the consequence of differences in the relative contribution of attention and prediction processes. The model's predictions will be discussed in the context of a functional interpretation of the auditory N1 as an attention call signal, as presented in a recent model on auditory processing.

## What is selective attention and how is it induced?

Our sensory systems are often exposed to a large amount of input from various external and internal sources. However, not all of this input is equally relevant to our current needs and goals. In order to deal efficiently with the resources at hand it is therefore adaptive to prioritize the processing of certain events. The process or set of processes by which prioritized processing is attained is referred to as attention. For example, Nobre (2004) referred to attentional orienting as “the set of processes by which neural resources are deployed selectively toward specific attributes of events on the basis of changing motivation, expectation, or volition in order to optimize perception and action. Operationally, orienting can be measured through the behavioral consequences of changes in stimulus salience, predictability, or relevance” (p. 157). In addition to addressing the idea of prioritized or exclusive processing, this definition points out that attention can be oriented to various attributes of a stimulus event (e.g., spatial position or pitch) and that there are different ways to operationalize attention: manipulating stimulus salience, stimulus predictability, or stimulus relevance.

Stimulus predictability and stimulus relevance, respectively, are manipulated in two of the most frequently used paradigms in attention research: probabilistic cuing (e.g., Posner et al., 1980) and filter tasks (e.g., Cherry, 1953; Hillyard et al., 1973). In probabilistic cuing, stimulus predictability is manipulated, i.e., a symbolic cue indicates for instance the most likely spatial position of the upcoming target (e.g., Posner et al., 1980). In this task, behavioral benefits for predicted targets are attributed to an orienting of attention to the expected position (because, on average, this will be beneficial for task-performance). By contrast, in filter paradigms stimulus relevance is manipulated. For example, the spatial position of an event determines whether the event is relevant for performance, that is, whether it requires an overt (e.g., a key-press) or covert (e.g., counting) response (e.g., Cherry, 1953; Hillyard et al., 1973). Because stimuli at the other spatial location never require a response, it is assumed that processing resources are strictly focused on the task-relevant position. Stimuli presented at the relevant or irrelevant position are therefore regarded as attended or unattended, respectively.

Both probabilistic cuing and filter paradigms are associated with an orienting of attention. However, the effects that are measured in the two groups of tasks need not be identical, nor do they necessarily reflect identical (sets of) processes—including (but not restricted to) attentional processes. First, effects may be quantitatively different, i.e., attention effects may be larger in filter compared to cuing tasks. This is because in filter tasks only attended stimuli require a response, thus allowing for a strict focusing of attention. By contrast, in probabilistic cuing tasks, processing resources have to be (unevenly) *divided* between expected and unexpected stimuli, because both are associated with a response. Second, filter and cueing tasks may involve qualitatively different (sets of) processes. Cueing tasks first and foremost manipulate stimulus probability and hence participants' expectations. This is assumed to lead to an orienting of attention to the expected stimulus, similar as in filter paradigms. However, expectations may also exert effects on stimulus processing apart from those triggered by the orienting of attention: In other words “experimental results interpreted in terms of selective attention are often confounded by expectation effects” (Rauss et al., 2012, p. 1249). Although the conclusion that cuing tasks involve both expectations and attention may be partly obvious, it is not always acknowledged and addressed in attention research (for similar arguments see Summerfield and Egner, 2009; Nobre et al., 2012). An additional consequence of expectations is that participants may (successfully) engage in predictions as to which stimulus will be presented next. Because of this, the potential confounding of attention and expectations (and hence, predictions) may become problematic when pooling findings obtained in the different paradigms. Attention (i.e., the focusing of processing resources) and expectations or predictions are known to be associated with opposite effects on early event-related potentials (ERPs; see also Summerfield and Egner, 2009), particularly in the auditory modality (as detailed below). As a consequence, effects measured in scalp-recorded ERPs may differ as a function of the paradigm used—as is the case in studies related to the temporal orienting of attention (as detailed below). In the present manuscript, I will explain the different effects on the scalp-recorded ERP by assuming that both cuing and filter paradigms involve a focusing of processing resources (or orienting of attention), whereas only cuing paradigms involve processes related to stimulus prediction. To keep it simple, I assume that the attention process (the focusing of processing resources) is identical between the two paradigms (although this notion is surely debatable).

In the following, I will provide a brief overview on data corroborating the opposite effects of attention and predictability on brain correlates of early processing in the auditory modality, particularly the auditory N1 (paragraph 2). I will then discuss the problem to adequately assign the probabilistic cuing paradigm to one or the other category, although ERP data roughly resemble those obtained using filter paradigms (paragraph 3). I will then turn to the field of *temporal* orienting of attention in the auditory domain. I will start by reviewing recent ERP studies that manipulated expectations for and task-relevance of stimuli presented at particular points in time, respectively (paragraph 4), manipulations classically used to trigger an orienting of attention. The results of these studies with respect to the direction of the effect the experimental manipulation had on the auditory N1 were heterogeneous. Manipulations of task-relevance lead to increases of the N1 for stimuli at the task-relevant time point, whereas temporal expectations lead to increases, decreases, or null-effects for stimuli at the expected time point. One possibility is to ascribe this controversy to different sub-processes of attention that are involved in the two classes of paradigms. However, as an alternative, I will present a working model on the auditory N1 (paragraph 5) that is based on empirical findings that attention and prediction have opposite effects on early auditory ERPs (as reviewed in paragraph 2). The model assumes that paradigms manipulating (temporal) stimulus probabilities (such as probabilistic or rhythmic cuing) first and foremost trigger overall expectations for particular stimuli. These expectations are supposed to induce prediction processes known to decrease N1 amplitudes. Moreover, if stimuli require additional processing (e.g., because they are response relevant), expectations may also lead to a focusing of processing resources (i.e., an orienting of attention) to the expected stimuli. The latter process is assumed to correspond to the attentional orienting induced by task-relevance in filter paradigms, which is known to increase the auditory N1. Because of the opposite effects of these two processes, the net effect that can be measured in the ERP, depends on their relative contributions. The model is used to describe and explain the pattern of results of the existing auditory temporal orienting studies. Moreover, testable hypotheses can be derived of the model, which may encourage future research.

## Attention and stimulus predictability have opposite effects on early auditory ERPs

### Effects of attention based on task-relevance

Most ERP studies investigating auditory attention have used filter paradigms. In these paradigms, only stimuli sharing a certain feature (e.g., a certain spatial position) have to be evaluated with respect to their possible response relevance (e.g., Hillyard et al., 1973; Schwent et al., 1976; Näätänen et al., 1978; Giard et al., 1988; Woldorff and Hillyard, 1991). Hence, it is assumed that attention is strictly focused on the task-relevant channel, which is therefore regarded as attended. Attention effects measured in these tasks consist of enhanced negativities including an early Nd (peaking between 100 and 200 ms) with a fronto-central scalp topography and a late Nd (peaking around 300 and 400 ms) with a more anterior maximum (e.g., Näätänen et al., 1978; Hansen and Hillyard, 1980; Näätänen, 1982; Alho et al., 1987; see also Näätänen and Alho, 2004 for a review). The early Nd may also encompass a modulation of the sensory-evoked N1 (e.g., Hillyard et al., 1973; Giard et al., 1988; Rif et al., 1991; Alcaini et al., 1994; Ozaki et al., 2004). This may be regarded as evidence for a gating or filter mechanism of attention (e.g., Hillyard et al., 1973; Hillyard, 1981; Kauramaki et al., 2007; see also Hillyard et al., 1998), by which the processing of attended stimuli is favored over that of unattended ones.

### Effects of stimulus predictability

As opposed to attention, stimulus predictability is associated with a reduction (rather than an enhancement) of early negativities. Examples include the attenuation of the N1 elicited by auditory effects of one's own motor action (Schafer and Marcus, 1973; McCarthy and Donchin, 1976; Ford et al., 2001; Houde et al., 2002; Heinks-Maldonado et al., 2005, 2006; Martikainen et al., 2005; Bäß et al., 2008; Aliu et al., 2009; Lange, 2011) or by temporally predictable auditory stimuli (e.g., Schafer et al., 1981; Clementz et al., 2002; Ford et al., 2007; Lange, 2009; see also Vroomen and Stekelenburg, 2010)^1^.

The motor induced suppression of the N1 is typically explained by forward models of motor control (e.g., Miall and Wolpert, 1996; see also Sperry, 1950; Von Holst and Mittelstaedt, 1950). According to these models, whenever an action is initiated, predictions are made with respect to its sensory consequences. The actual outcome is then compared to the predicted effect: If both match, an attenuated response results. The suggested mechanisms are similar to what is suggested by the more general predictive coding framework (e.g., Friston, 2005): In a hierarchically organized sensory system, each level of the hierarchy receives both bottom–up, sensory information from the level below and top–down, predictive information from the level above. Deviations between sensory input and predictions cause an error signal, which is then propagated to higher levels to adjust the predictions. Assuming that the error signal is reflected in the scalp-recorded ERP, predictive coding can explain the reductions of negative ERP components associated with repetition suppression and sensory-predictable standards in oddball tasks: With improving predictions, the actual sensory input will more closely match top–down predictions, resulting in a smaller error signal—and hence, a reduction of the stimulus-evoked ERPs (Baldeweg, 2007). Given the similarity between ERP attenuations observed with sensory predictions and the motor-related ERP suppressions, it seems plausible to assume that similar predictive mechanisms may be applied when deriving predictions from external stimulation and from internal motor commands (for similar arguments see also Schubotz, 2007 and Sowman et al., 2012). Hence, motor- and sensory predictions may constitute different sources for a single mechanism implementing top–down predictions in perceptual processing.

## Probabilistic cuing: caught in the middle between attention and predictability

Although probabilistic cuing is typically regarded as a manipulation of attention (e.g., Mangun and Hillyard, 1991), studies using this paradigm cannot be unequivocally classified with respect to the dichotomy between attention and predictability: On the one hand, cued and uncued stimuli occur with different probabilities, i.e., these studies manipulate stimulus predictability. On the other hand, in a typical probabilistic cuing task, all stimuli require an overt response. It is therefore highly adaptive to orient attention to the most probable—hence expected—event: On average, this will yield the highest performance. Therefore, the expected event also becomes the attended event (though presumably to a lesser degree than in filter tasks). At the same time, it is reasonable to assume that expectations are used to generate (more or less) precise predictions as to which stimulus will be presented next—similar to the predictions based on internal forward models described above. It may thus be concluded that probabilistic cuing tasks confound attention and expectation/prediction (see also Summerfield and Egner, 2009; Kok et al., 2012; Rauss et al., 2012). In spite of this confound, results obtained in ERP studies employing probabilistic cuing are similar to those measured in filter paradigms (e.g., Schröger, 1993, 1994; Schröger and Eimer, 1997; see also Mangun and Hillyard, 1991 for visual results). For example, Schröger (1993) reported a similar early negativity of transient auditory attention using both a pure filter task (Experiment 1) and a filter task combined with probabilistic cuing (Experiment 2). Assuming that probabilistic cuing induces both an increase in stimulus predictability and an orienting of attention and assuming additive effects of these processes on the auditory N1, the observable ERP effect might reflect the fact that the attention-related enhancements outweighed the reductions induced by stimulus predictability. Hence, the direction of the probabilistic cuing effect on the auditory ERP may depend on the different factors that contribute to the orienting of attention on the one hand and to event predictability on the other. Consistent with this notion, at a descriptive level, the attention effect in the early negativity observed by Schröger (1993) was smaller when a filter task was combined with probabilistic cuing (Experiment 2; i.e., both attention and prediction are involved) compared to the use of a pure filter task (Experiment 1; only attention is induced). Findings that are consistent with this notion are also obtained in the field of auditory temporal attention, as detailed below.

Note, however, that additivity of attention and prediction is not the only possibility. A recent application of predictive coding theory to probabilistic cuing explains enhanced ERPs to attended stimuli in the Posner-task by assuming *synergistic* effects of attention and prediction. According to this notion, attention is supposed to boost the precision of prediction, thus leading to a heightened weighting of sensory evidence (Feldman and Friston, 2010). This is supposed to reverse effects of “sensory silencing,” and hence leads to the typical amplitude increase in ERPs to attended stimuli in probabilistic cuing tasks (see also Kok et al., 2012). Predictive coding models have been successfully applied to explain the enhanced ERPs observed in (spatial) probabilistic cuing in the visual domain (e.g., Mangun and Hillyard, 1991) and, moreover, the predictions of these models have been corroborated by recent functional Magnetic Resonance Imaging evidence (Kok et al., 2012).

## Temporal orienting of attention

In recent years, a growing number of studies investigated the temporal orienting of attention, i.e., the selection of information for prioritized processing based on the time of stimulus occurrence (e.g., Nobre and Coull, 2010). Similar to spatial attention, temporal attention has been induced by manipulating the task-relevance of stimuli at particular time points or the expectations for stimuli at particular time points. However, whereas in the spatial domain, enhancements of early ERP components are a common finding for orienting based on both task-relevance and on expectations, these two paradigms yield somewhat different results when it comes to the temporal orienting of attention.

### ERP studies of temporal orienting based on task-relevance

**Figure 1:**
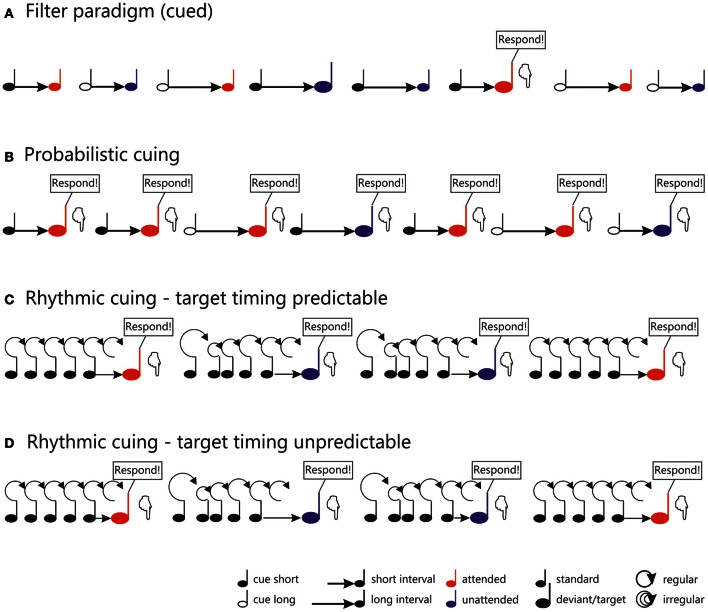
**General outlines of stimulus configurations used in different temporal orienting tasks.** In the cued filter paradigm **(A)**, as used e.g., in Lange (2012a), each trial consists of a symbolic cue (black note symbol) indicating whether the short (filled) or the long (unfilled) interval has to be monitored for deviant sounds. Frequent standard sounds (small note symbols) and infrequent deviantssounds (large note symbols) follow the cue after a short or long interval (indicated by the length of the arrow). Standards and deviants presented at attended and unattended time points are marked by red (attended) and blue (unattended) note symbols, respectively. In this paradigm, only attended deviants require a response, as indicated in the figure. In the probabilistic cuing paradigm **(B)** as used in Lampar and Lange (2011, Experiment 1), the symbolic cue (black note symbol) indicates the time point, when the target will most likely be presented: Either at the end of the short (filled) or at the end of the long (unfilled) interval. In this paradigm, sounds presented at both attended (red) and unattended (blue) time points require a response and are therefore regarded as targets (large note symbols) **(C,D)**. In the rhythmic cuing task, a regular or irregular induction sequence (black note symbols connected by circles of fixed or variable size) is presented prior to the target (indicated by a red or blue note symbol, depending on whether it is preceded by a regular or an irregular sequence). In the task displayed in **(C)** (Lange, 2009), the timing of the target with respect to the sequence was fixed (indicated by the identical length of the arrows connecting the sequence to the target), whereas target timing varied in the task outlined in **(D)** (Lange, 2010), as reflected in the variable length of the arrows.

Most of the studies inducing temporal attention by manipulating stimulus relevance used a variant of the selective attention paradigm introduced by Hillyard et al. (1973). In the temporal version of this paradigm, two sounds are presented in each trial, which are separated by a shorter or longer temporal interval (e.g., 600 vs. 1200 ms; Lange et al., 2003; see also Figure 1A). The first sound is a cue and marks the onset of the interval. The second sound is either a frequent standard stimulus or an infrequent deviant stimulus (e.g., louder or softer than the standard, Lange et al., 2003, 2006). Participants are asked to respond to the deviants, but only if they follow the cue after a specified time interval, marking the attended time point. Sounds presented at the other time point never require a response and can be completely ignored. Which time point is attended is either indicated prior to each block of trials (for a review see Lange and Röder, 2010) or is signaled trial-by-trial by the nature of the cue (Lange, 2012a,b). In this paradigm, only stimuli at the cued time point require further evaluation and categorization as standard or deviant. Thus, processing resources are likely dedicated predominantly to this time point, and it is regarded as attended. Because attended but not unattended deviants require an overt response, it is important to control for motor confounds. For this reason, ERPs to standard stimuli are used to measure attention effects, because standard stimuli do never require an overt response.

**Figure 2:**
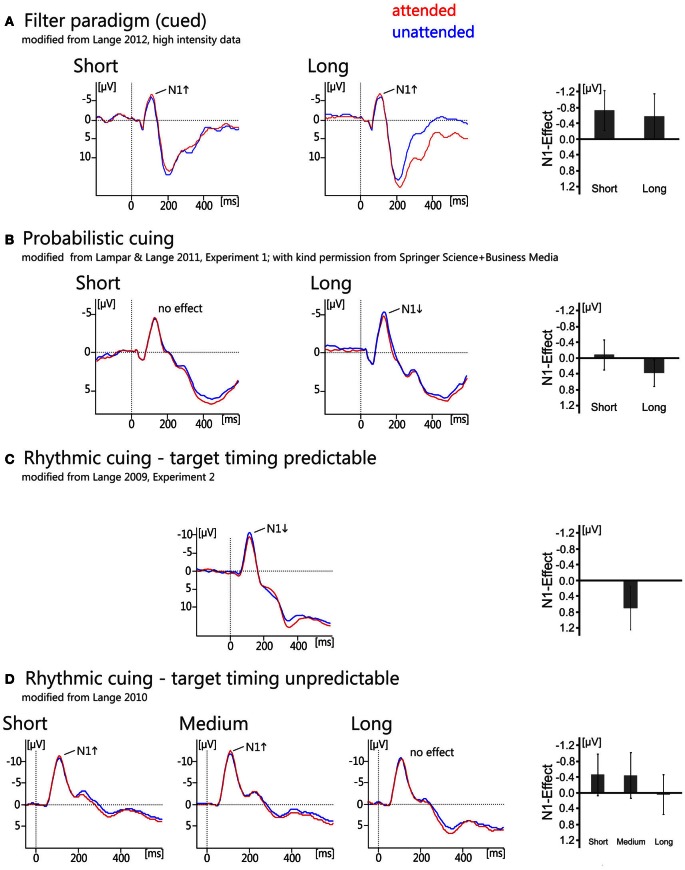
**Overview of grand average ERP waveforms (left) and bar graphs of the effects (right) showing the N1-effects of temporal orienting as measured in temporal filter tasks (A), temporal probabilistic cuing (B), and rhythmic cuing with and without predictability of target timing (C,D).** For the filter task **(A)** and probabilistic cuing **(B)**, ERPs are shown for electrode Cz and C3, respectively, separately for the short and the long interval. Traces are aligned to a post-stimulus baseline from 0 to 50 ms. For the rhythmic cuing task with predictable target timing, the effect is depicted at electrode Cz and traces are aligned to a 200 ms pre-stimulus baseline. For the rhythmic cuing task with unpredictable target timing, the effect is depicted at a left electrode cluster consisting of electrodes Fz, Cz, and Pz, and traces are aligned to a 0–50 ms post-stimulus baseline. Bar graphs show the attention effects (ERP_attended_ minus ERP_unattended_), with error bars representing the 95% confidence limen.

Most of the studies using this approach in the domain of temporal orienting have employed auditory stimuli and found evidence that temporal attention operates early in the processing chain, as evidenced by an enhancement of the auditory N1 around 100 ms post-stimulus (see Figure 2A; Lange et al., 2003, 2006; Lange and Röder, 2006; Röder et al., 2007; Sanders and Astheimer, 2008; Lange, 2012a,b; see also Chait et al., 2010 for related data; but see Griffin et al., 2002, Experiment 2 for visual data suggesting later effects). Hence, the data obtained with filter paradigms consistently show that temporal orienting modulates early auditory processing as reflected in the amplitude enhancement of the auditory N1.

### ERP studies of temporal orienting based on temporal expectations

**Figure 3:**
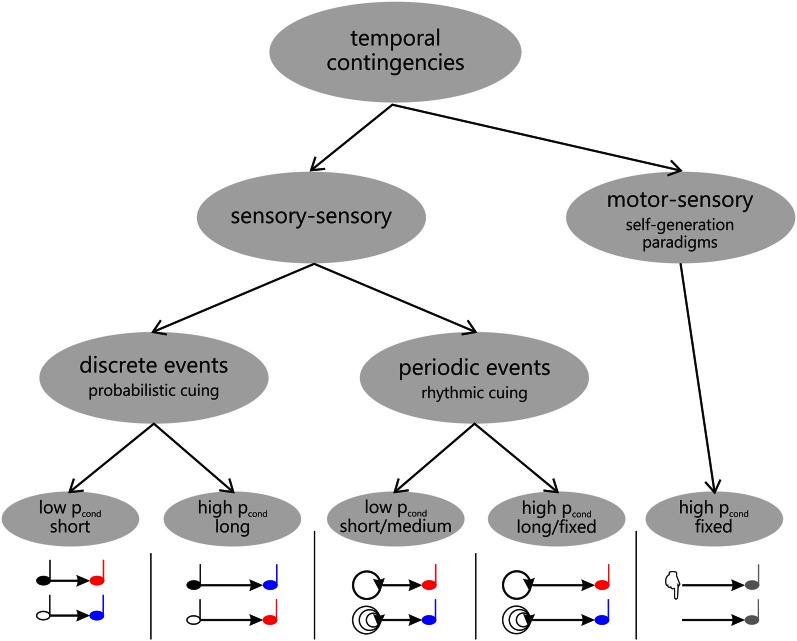
**Overview of different types of contingencies underlying temporal expectations as manipulated in probabilistic (left) and rhythmic cuing (middle) and self-generation paradigms (right), respectively**.

Expectations are derived from contingencies (or probabilistic relationships) between events, as experienced in the environment. These contingencies may not only include information relating to which event is about to occur, but also relating to when a particular event is to be expected. Within this context, the studies investigating motor induced suppression of sensory processing (reviewed above) may be characterized as establishing a temporal contingency between motor acts and sensory events (as illustrated in Figure 3, right). However, temporal relationships may also be established between separate sensory events. As for these sensory-sensory contingencies, it seems useful to further distinguish between discrete and periodic events (Figure 3, left and middle), which are used to induce expectations in probabilistic and rhythmic cuing paradigms, respectively (probabilistic cuing: e.g., Coull and Nobre, 1998; Miniussi et al., 1999; Correa et al., 2004, 2005; see also Correa et al., 2006; Lampar and Lange, 2011, Experiment 1; rhythmic cuing: e.g., Doherty et al., 2005; Lange, 2009, 2010)^2^.

A specific feature of expectations in the time domain calls for a further distinction: The fact that the flow of time itself provides information as to whether an event is about to occur. The conditional probability that an event will occur at a particular time point given that it has not yet occurred increases as a function of time, being low directly after the cue has been presented and approaching certainty for the latest possible time point, when an event may occur. This relationship is referred to as the hazard function and it is known to affect both reaction times (for a review see Niemi and Näätänen, 1981) and brain activity (single-cell recordings e.g., Riehle et al., 1997; Ghose and Maunsell, 2002; Janssen and Shadlen, 2005; Schoffelen et al., 2005; Ghose and Bearl, 2010; functional imaging data: Bueti et al., 2010). Although most ERP studies investigating the temporal orienting of attention have not addressed this issue explicitly (for an exception see Correa and Nobre, 2008), it seems worth acknowledging the difference between low vs. high conditional probability when reviewing existing findings on temporal orienting research in order to explain the different effects^3^.

#### Temporal relationships between discrete sensory events: probabilistic cuing

Temporal probabilistic cuing is a temporal variant of the Posner-cuing task (Posner et al., 1980). In the temporal version, a symbolic cue indicates the time point when the target is most likely to be presented (Figure 1B). In most studies, two different time intervals are randomly cued, a short and a long interval (e.g., 600 and 1400 ms, Miniussi et al., 1999). The target appears with a high probability at the end of the cued interval and with a low probability at the end of the other interval. While a behavioral benefit is commonly observed using this kind of task (for a review of visual data see Correa, 2010; for auditory data see also Lampar and Lange, 2011 and the behavioral experiment reported in Lange and Röder, 2006), it remains an open question whether or not temporal probabilistic cuing operates on the same processing stages as temporal attention based on task relevance. As noted above, the flow of time itself provides information with respect to whether a stimulus will be presented. Hence, when the short interval is cued but the interval is actually long (i.e., no stimulus appears at the end of the short interval), the time of stimulus presentation becomes certain. In this case, when a response is required regardless of attention—as in probabilistic cuing—it is adaptive to re-orient ones attention to the other (i.e., the long) interval, leading to similar processing resources dedicated to the ending of the long interval in attended and unattended conditions. Because of this, behavioral effects (consisting of faster responses to temporally attended stimuli) are typically restricted to stimuli presented after a short interval (e.g., Coull and Nobre, 1998; Miniussi et al., 1999; Griffin et al., 2001, 2002; Correa et al., 2004, 2006). The associated ERP findings are heterogeneous, however: Some studies do not find any evidence that temporal attention affects early, sensory ERP components for the short interval (visual: Miniussi et al., 1999; auditory: Lampar and Lange, 2011, Experiment 1; see also Figure 2B, left) or found an enhancement, but in a later component than observed in spatial attention (visual: Griffin et al., 2002, Experiment 1). Only Correa et al. (2006), who manipulated expectations in a block-wise manner, reported an enhancement of the sensory P1 component of the visual ERP around 100 ms post-stimulus. As for the long interval, where behavioral effects are typically not observed (but see Griffin et al., 2002, Experiments 1 and 2, where more than two intervals were used to overcome the problem of temporal predictability for later than expected stimuli), ERPs seemed to be unaffected by temporal attention in the visual domain. By contrast, there is evidence that the auditory N1 to attended stimuli of the long interval is reduced by temporal attention (Figure 2B, right; Lampar and Lange, 2011, Experiment 1). A potential explanation relates this effect to the interplay between a priori and conditional probability, which differs between the short and the long interval. This idea will be detailed below. To summarize, existing ERP studies paint a heterogeneous picture with respect to the potential effects of expectancy-based temporal attention on early, sensory processing.

#### Temporal relationships between periodic sensory events: rhythmic cuing

In the time domain, we do not only establish expectations by assessing the probabilities of particular temporal delays between discrete stimuli as in probabilistic cuing. Being exposed to a temporally regular, repetitive sequence of stimuli such as the ticking of a clock or the flashing of a turning signal, we expect the pattern to continue and may thus anticipate the next tick of the clock or the next flashing of the light. Several recent ERP studies investigated the impact of a temporally regular stimulus sequence on the processing of subsequent stimuli. Most studies compared stimulus processing between conditions where the target followed a regular vs. an irregular sequence (e.g., visual: Doherty et al., 2005; see also Rohenkohl and Nobre, 2011; auditory: Lange, 2009, 2010; Rimmele et al., 2011), or between conditions where stimuli followed regular sequences of different tempi (auditory: Sanabria and Correa, 2013; see also Correa and Nobre, 2008).

***Rhythmic cuing in the auditory domain***. The susceptibility of early auditory processing to the temporal orienting of attention has been demonstrated by several studies manipulating temporal attention by means of task-relevance (Lange and Röder, 2010). Hence, it came at no surprise that rhythmic cuing in the auditory domain was also associated with early, sensory effects (Lange, 2009, 2010). However, the direction of the early effect varied with the specific experimental settings: Whereas a reduction of the N1 was observed in the two experiments reported in Lange (2009), an enhancement was found in a later study (Lange, 2010).

Lange (2009) presented temporally regular or temporally irregular tone sequences prior to a target tone (Figure 1C; see also Doherty et al., 2005, for a visual version of this task). The tones of the each sequence were presented either as a scale (ascending or descending; predictable pitch) or the pitches of the sequence tones varied unpredictably. The target tone followed the sequence after an interval equivalent to the omission of two steps of the regular condition. Faster responding was observed in the regular compared to the irregular condition (similar to the visual study of Doherty et al., 2005). Analysis of the ERP data showed that valid temporal expectations were associated with an amplitude attenuation in the time range around 100 ms, i.e., a reduction of the auditory N1, compared to the condition, where *no* expectation was induced (Figure 2C; but see Rimmele et al., 2011, who found an enhancement of the N1 with a similar manipulation). Crucially, because a valid expectation condition was compared to a condition without any expectation, the observed effect can be distinguished from the family of mismatch responses (for a review see Schröger, 1998), which mainly reflect response to expectancy violations. Additionally and consistent with other findings on rhythmic cuing (Doherty et al., 2005), temporal expectations also enhanced the P3 (see also Correa and Nobre, 2008; Rohenkohl and Nobre, 2011).

***The role of temporal predictability in rhythmic cuing***. The reduction of the auditory N1 to rhythmically expected sounds (regarded as attended in the rhythmic cuing paradigm) contrasts with findings of earlier auditory temporal orienting studies, which reported enhancements of the N1 (for a review see Lange and Röder, 2010). Because of the opposite polarities of the ERP effects, one may assume that manipulations of the two paradigms (rhythmic cuing and filter tasks) reflect separable attention processes. Hence, the reduction of the N1 might constitute a specific correlate of what may be termed rhythmic attention whereas the enhancement of the N1 might be specific to attention based on task-relevance. There is, however, an alternative explanation, which is compatible with the assumption of a single attention process with a uniform effect on stimulus processing: The reduced N1 could have reflected the increased predictability of stimuli in the rhythmic compared to the arrhythmic condition. Sensory predictability is also known to be associated with attenuated N1 amplitudes (e.g., Schafer et al., 1981; Clementz et al., 2002). In Lange (2009), the final interval was of equal duration in the rhythmic and in the arrhythmic condition to eliminate the possibility of using top–down knowledge of the last interval in the rhythmic but not the arrhythmic condition. Hence, in both conditions participants were able to predict exactly when the final sound would occur. However, the estimation of an interval benefits from its frequent presentation (e.g., Drake and Botte, 1993). Therefore, prediction might have been particularly precise when the sequence was regular, because here the same interval is frequently presented. By contrast, in studies inducing an orienting of temporal attention by manipulating task-relevance, the time point of target presentation is not predictable at the onset of a trial (for a review see Lange and Röder, 2010). The fact that these studies consistently observed an increased N1 to temporally attended stimuli, whereas rhythmic cuing was associated with an amplitude decrease (Lange, 2009) might thus be due to differences in temporal predictability rather than fundamental differences between different ways to manipulate temporal orienting.

A follow-up study (Lange, 2010) corroborated the notion that the N1 attenuation obtained in Lange (2009) may have been due to increased temporal predictability in the rhythmic condition. This study used basically the same paradigm as Lange (2009), i.e., a regular or an irregular sequence was presented prior to a target tone. However, the new design of Lange (2010) also included targets at time points earlier or later than the time point marked by the rhythmicity of the sequence (Figure 1D). Hence, the sequence could *not* be reliably used to predict the timing of target onset (which had been possible in the 2009 study). Notably, N1 to rhythmically attended stimuli was no longer attenuated in the regular compared to the irregular condition, which is consistent with the notion that the N1 attenuation observed in the earlier study (Lange, 2009) was due to temporal prediction processes rather than rhythmic attention (see also Vroomen and Stekelenburg, 2010 for similar results). Interestingly, a small but reliable enhancement of the N1 was found for the rhythmic compared to the arrhythmic condition in Lange (2010)^4^. This effect is consistent with earlier findings of an enhanced N1 in auditory temporal attention studies (Lange and Röder, 2010) and may thus reflect an orienting of attention in time.

***Rhythmic cuing may affect stimulus processing both by prediction and by attention***. Further analyses showed that the N1 enhancement was only observed for stimuli in the short and medium interval condition, whereas no effect (a small reduction at the descriptive level) was found for auditory targets presented after the longest interval (Figure 2D). Notably, for the long interval stimulus occurrence is certain due to conditional probability. Hence, this pattern of results suggests that prediction processes affected stimulus processing even in the paradigm used by Lange (2010)—when considering the contribution of conditional probability to overall predictability, as already suggested for probabilistic cuing (Lampar and Lange, 2011, long interval data).

It may therefore be hypothesized that presenting a rhythmic sequence triggers two processes with opposite effects on stimulus processing: The first is similar to what is manipulated when task-relevance is used to induce an orienting of attention and leads to an enhancement of the N1. This process dominates when target timing is uncertain because of reduced a priori probability and/or reduced conditional probability, leading to the N1 enhancement in the short interval and medium interval conditions of Lange (2010). The second process is related to the increased predictability of stimulus onset in the rhythmic condition and leads to a reduction of the N1. This process may dominate the ERP effect when the sequence can be reliably used to predict the moment of the sound's onset—either due to the fixed a priori probability (as in Lange, 2009) or because of an increased conditional probability (as in Lange, 2010, long interval; see also Lampar and Lange, 2011, long interval). Predictions may depend on the interplay between conditional probability (that stimulus occurrence becomes more and more likely with elapsing time), a priori probability (that the final interval will take a particular value), and rhythmic expectations (that the regularity of the sequence will be continued).

## Explaining effects of auditory temporal orienting by opposite effects of attention and prediction

Summarizing the core findings, the ERP effects measured in studies operationalizing temporal attention (i.e., the focusing of processing resources to a point in time) by manipulating task-relevance and expectations, respectively, are not identical: Studies operationalizing temporal attention through task-relevance consistently report enhancements of early, sensory ERP components, whereas studies manipulating temporal expectations yield mixed results, showing either enhancements or attenuations of early, sensory processing, or no effects. Studies using filter paradigms primarily focused on auditory stimuli, whereas probabilistic and rhythmic temporal cuing have been employed both in vision and in audition. Notably, results are not less heterogeneous when considering only the auditory studies, suggesting that the discrepant findings on early, sensory effects are not due to stimulus modality—although the precise role of modality still needs to be explored. Given the pattern of results of the studies reviewed above, another explanation seems likely: The discrepancies in the ERP effects may result from the fact that the increased expectations based on regularity and probability manipulations not only induced an orienting of attention to the expected point in time, but—at the same time—induced processes of predicting stimulus onset that may have counteracted the enhancing effect of attention on N1.

In the following, I will present a working model that describes N1 amplitude as a function of a single attention process on the one hand and a prediction process the other. Assuming that previous temporal orienting studies involved processes of attention and prediction to different degrees, this model can explain most of the partly divergent findings of previous studies with respect to the auditory N1. Moreover, it leads to novel predictions concerning the interplay between attention and prediction.

### A working model on N1 effects in tasks related to temporal attention

**Figure 4:**
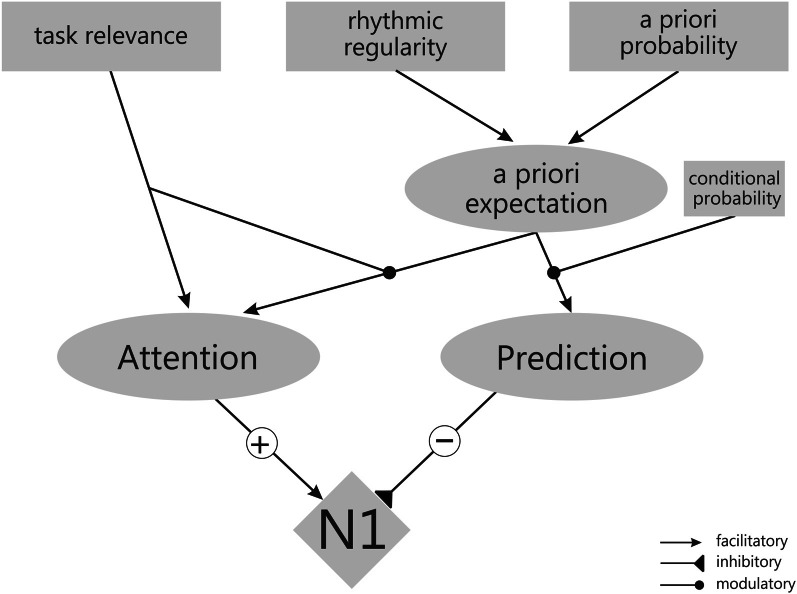
**Schematic outline of the components (gray boxes) and relations (black lines) assumed by the working model explaining N1 amplitude by opposite effects of attention and predictability**.

Figure 4 depicts the main components of the model and how they might relate. The model assumes that attention (Figure 4, left) and temporal prediction (Figure 4, right) have opposite effects on the amplitude of the N1: Attention leads to an enhancement of the N1 (hence the positive (+) influence of Attention] and prediction to an attenuation [hence the negative (−) influence of Prediction; see Equation 1].

(1) N1=Attention−Prediction

The orienting of attention refers to the allocation of processing resources. The orienting of attention may rely only on task-relevance (as in filter tasks), being independent of any stimulus expectations (hence the additive component γ in Equation 2). Additionally, the allocation of processing resources may follow ones (temporal) a priori expectation that a stimulus will occur at a particular point in time (Equation 2). This depends on the a priori probability that a stimulus occurs at a particular time point relative to another event (i.e., the relative proportion of trials with a given temporal relationship between cue and a target, as in temporal probabilistic cuing), and on the overall degree of temporal regularity in the sequence of stimuli (i.e., periodic stimulus presentation, particularly in isochronous sequences, as in rhythmic cuing). Because the rhythmic regularity of a sequence is experienced almost instantaneously whereas probabilistic features are extracted only after considering a larger number of trials, it is proposed that rhythmic cuing has a stronger influence on a priori expectations than a priori probability (i.e., β > 1, see Equation 3). Most importantly, however, it is assumed that attention will only be focused to the expected time point, when stimuli at this time point are relevant for task performance—because only in this case (additional) resources are needed for stimulus processing (hence the multiplicative relation between task-relevance and expectation in Equation 2). Finally, according to the model, the orienting of attention is triggered only at the onset of a trial and does not follow the increasing conditional probability during the course of the trial (in other words, conditional probabilities do not influence the orienting of attention).

(2) $$\begin{array}{l} \text{Attention}=\text{task-relevance}\\ \;\times (\text{a priori expectation}+\gamma ) \end{array}$$

(3) $$\begin{array}{l} \text{A priori expectation}=\text{a priori probability}+\beta \\ \;\times \text{ rhythmic regularity} \end{array}$$

Both in cuing paradigms and in filter paradigms participants are asked to (more or less) frequently respond to targets. Because this requires a certain amount of processing resources, it is reasonable to assume that participants use any information available to most efficiently allocate their processing resources (or orient their attention)—either because a subset of stimuli is expected (as in cuing paradigms) or because only a subset of stimuli requires deeper processing at all (as in filter paradigms). Hence, it is assumed that probabilistic cuing (Lampar and Lange, 2011), rhythmic cuing (Lange, 2009, 2010), and filter paradigms (e.g., Lange et al., 2003; Sanders and Astheimer, 2008) should all involve an allocation of processing resources to a subset of stimuli, and hence attentional differences between conditions. The model assumes a single attention process to be involved in cuing and filter tasks. Note, however, that it is also conceivable in principle that filter and cuing tasks involve qualitatively different mechanisms. Whether one has to distinguish selection-based and expectation-based temporal attention, and whether these involve only strategic or also automatic processes remains open. Further research is needed to further explore the precise nature of the attention process (or processes) involved. Importantly, the fact that targets are relevant for response selection seems to be crucial for orienting of attention, i.e., the mere presence of expectations *alone* should not lead to an orienting of attention. For example, in self-generation paradigms expectations about action effects are generated, but these action effects typically do not require any response. Here, a differential orienting of attention compared to the control condition should not automatically be induced (for a discussion of this point see also Lange, 2011).

The second major component of the model is temporal prediction (Equation 4). Temporal prediction is a direct consequence of (1) the a priori expectation (or global expectation; specified in Equation 3) and (2) the conditional probability (or local expectation) that a stimulus will occur at a particular time point (i.e., the hazard rate).

(4) $$\begin{array}{l} \text{Prediction}=\text{a priori expectation}\\ \;\times \;(\alpha \times \text{conditional probability}) \end{array}$$

Increasing a priori expectations—either based on increases in the a priori probability for a stimulus at a particular time point (e.g., for valid stimuli in probabilistic cuing or for stimuli following one's own motor action) or by presenting the stimulus as part of a rhythmically regular sequence (as in rhythmic cuing)—not only lead to a biased allocation of processing resources as outlined above. They also allow more or less precise prediction of when the next stimulus is about to occur. The possibility to precisely predict the moment of stimulus occurrence is, however, not only dependent on a priori expectations, set up at the beginning of a trial, but also on the conditional probability. When events are presented with a uniform (or “aging”) distribution over time—as in the studies cited above—conditional probability relates to the increasing certainty that the stimulus will occur “right now” the more time elapses. In this case, participants can more and more precisely predict the point in time, when the stimulus is about to occur. The model assumes that the conditional probability has a modulating influence on a priori expectations, i.e., its impact on prediction is only observed when there are differences in either a priori probability or rhythmic regularity. However, the influence of conditional probability on prediction is assumed to be stronger than that of the other two components (i.e., α > 1).

According to these assumptions, in a probabilistic cuing task, invalid stimuli (low a priori probability) following the cue after a short interval (low conditional probability) are particularly unpredictable, whereas valid stimuli (high a priori probability) following after a long interval (high conditional probability) are particularly predictable (e.g., Lampar and Lange, 2011). Likewise, stimuli presented as part of a periodic sequence are more predictable than stimuli presented as part of a random sequence—particularly when they match the regularity of the sequence (e.g., Lange, 2009). Finally, because of learned contingencies, sounds triggered by one's own motor act can be predicted more precisely than externally triggered sounds, since predictability of the key-press is already increased (e.g., Lange, 2011, see also Hughes et al., 2013).

The model is consistent with effects observed in temporal probabilistic cuing (no effect on the auditory N1 for the short interval and a reduction for the long interval; Lampar and Lange, 2011, Experiment 1), the reduced N1 to self-generated stimuli in the self-generation paradigm; Lange (2011), and the reduced N1 in rhythmic attention when the rhythmic sequence reliably predicts sound onset (Lange, 2009). For rhythmic attention with uncertainty, the model as presented here predicts an enhancement for the rhythmic condition that changes to a reduction over time, which is also similar to the pattern observed in Lange (2010). Finally, the model adequately describes the N1 enhancements obtained in filter paradigms (e.g., Lange et al., 2003, 2006; Sanders and Astheimer, 2008). The only temporal orienting data that cannot be easily described by the model *as is* are the results for the long interval of Lampar and Lange (2011), Experiment 2. This experiment has elements of both a probabilistic cuing task (attended stimuli are more likely than unattended ones) and of a filter task (responses are only required for attended stimuli). The model predicts an enhancement of the N1 for both the short and the long interval. However, the pattern observed is consistent with this prediction only for the short interval. For the long interval, the opposite effect was observed. Therefore, further studies are needed to identify limiting conditions or complement the model by further variables and/or further relationships between variables.

Notably, the model also gives rise to several novel predictions for effects of task-relevance, rhythmic or probabilistic cuing, and motor-induced predictions—when different tasks are combined. First, according to the model, manipulations of task-relevance should yield smaller effects on N1 when combined with rhythmic or probabilistic cuing. This is because rhythmic or probabilistic cuing increases predictions for attended stimuli: Hence, the enhancing effect of attention on the N1 will be counteracted by the decreasing effect of (valid) predictions. Because, however, both cuing manipulations are thought to increase *both* attention *and* prediction, the reduction of the N1 effect may be relatively small. Hence, one may have to make an effort to demonstrate this empirically. Notably, however, at a descriptive level, there are findings which are well in line with this assumption: In the spatial attention study by Schröger (1993), attention effects on N1 were larger when attention was manipulated by task-relevance alone (Experiment 1) compared to a condition, in which task-relevance and probabilistic cuing were combined (Experiment 2). Moreover, in previous studies of temporal orienting, the N1 effects seemed to be more pronounced in studies employing pure manipulations of task-relevance (e.g., Lange et al., 2003; Sanders and Astheimer, 2008) compared to a combination of task-relevance and probabilistic cuing (Lampar and Lange, 2011). Second, both rhythmic and probabilistic cuing may consistently lead to a reduction of the N1, when only passive stimulation is used. This is, because the model assumes that the impact of rhythmic and probabilistic cuing on attention depends on the (potential) task-relevance of these stimuli. In this case, the probability manipulations will not induce an orienting of attention, while predictions are still possible—similar to earlier studies of sensory predictability or studies of motor-induced suppression (e.g., Schafer and Marcus, 1973; McCarthy and Donchin, 1976; Schafer et al., 1981; Ford et al., 2001, 2007; Clementz et al., 2002; Bäß et al., 2008). In a related fashion, the model also predicts that the motor-induced suppression of the N1 to the effects of one's own actions may be *reduced* if participants oriented their attention to the self-elicited stimuli because they were relevant to the task at hand. In this case, stimuli rendered predictable by means of the preceding motor action would not only elicit a prediction-related decrease but in addition an attention-related increase of the N1, resulting in a reduction (or even an elimination) of the overall effect. Future studies are needed to address these hypotheses and provide evidence in favor of the basic idea represented in the model. Moreover, future research may investigate how the attention and prediction processes are related when it comes to non-temporal stimulus features.

### Open questions

Although the physiological mechanisms and the functional interpretation of the heterogeneous N1 effects still need to be identified, I will briefly discuss a tentative account of a potential physiological mechanism and a functional interpretation in the following. It is conceivable, that both the N1 attenuation induced by stimulus predictability and the N1 enhancement induced by attention reflect a modulation of the frontal subcomponent 3 of the auditory N1 (according to Näätänen and Picton, 1987). Näätänen and Picton (1987) already discussed the notion that the frontal sub-component of the N1 is attenuated by temporal predictions (“knowledge of the timing of the stimulus,” p. 412) and recent studies suggest that this unspecific component is also involved in motor-induced suppression of the N1 (SanMiguel et al., 2013; Timm et al., 2013). Notably, it has been suggested recently that this component may also be subject to manipulations of temporal attention (Lange, 2012a).

The processes underlying the N1—particularly its frontal sub-component—are most likely not involved in the perceptual analysis and the identification of specific sound attributes (e.g., Davis and Zerlin, 1966; Parasuraman and Beatty, 1980; Butler, 1972; Pratt and Sohmer, 1977, see Näätänen and Picton, 1987 for a review). Rather, there is evidence that the amplitude of the N1 is related to the detection of the onset of a sound (e.g., Davis et al., 1968; Squires et al., 1973; Parasuraman and Beatty, 1980; Parasuraman et al., 1982) and to the sound's attention-catching properties, more distracting sounds being associated with a larger N1 (Campbell et al., 2003; Rinne et al., 2006). Acknowledging these and other findings, Näätänen et al. (2011) suggest that the N1 is associated with an attention call signal, triggered by a mechanism dedicated to onset-detection. According to these authors, early auditory processing engages two parallel pathways: One dedicated to onset detection and one associated with auditory feature analysis. It is assumed that the N1 (particularly the frontal sub-component) is generated by the detection mechanism and that the main function of the underlying process is to increase the likelihood that the outcome of the feature analysis mechanism becomes available for conscious perception (e.g., Näätänen et al., 2011).

The next step for future studies is to identify the precise physiological mechanisms behind the ERP effects of attention and predictability. If temporal attention and temporal predictions indeed modulate the frontal sub-component of the N1 (e.g., Näätänen and Picton, 1987; see also Lange, 2012a), their respective functional roles could be to enhance and reduce the attention-catching properties of sounds and hence the likelihood of conscious sound processing. From a functional point of view, such an interpretation is highly plausible: An increased attention call for task-relevant sounds is adaptive, since these stimuli typically require an overt response, hence necessitating further conscious processing and evaluation. By contrast, in studies investigating pure effects of sound predictability, processing requirements are mostly similar for predictable and unpredictable sounds. In this case, there is less need for a mechanism promoting differential conscious processing. Moreover, when thinking of the most common instance of predictable events, i.e., all kinds of sensory events that result from our own motor actions, it is even more adaptive to reduce the likelihood of conscious processing—otherwise we would be almost constantly distracted by what seem by-and-large irrelevant events.

## Conclusions

In the present paper, I briefly reviewed the heterogeneous ERP data of auditory temporal orienting paradigms using either task-relevance or expectations to induce a temporal orienting of attention. In order to explain this pattern of results, I presented a working model assuming that both manipulations activate a single attention process that enhances the auditory N1. Paradigms manipulating expectations to induce attention additionally involve prediction processes, which lead to an N1 attenuation. Open questions that are of relevance with respect to the interpretation of the enhancing and reducing effects of attention and predictions on N1 amplitudes concern the physiological mechanisms underlying these effects and their functional significance. With respect to the physiological interpretation, it needs to be investigated whether prediction and attention affect the same process in opposite directions—or whether they merely co-occur in time. Future studies may address this question by employing orthogonal manipulations of attention and prediction in the same experiment to test whether or not the respective effects are additive. Moreover, the physiological mechanisms underlying these effects need to be identified: Do they constitute modulations of the sensory-evoked N1 (or one of its subcomponents) or are they due to additional, endogenous voltage shifts (e.g., Giard et al., 2000 for a review on a similar discussion for non-temporal attention). The answer(s) to this question will also help to pinpoint the functional interpretation of the effects. Finally, the working model as presented here is not meant to be exhaustive. Rather, it is a first approach to explain most of the partly discrepant findings of previous temporal orienting research in a parsimonious way by taking into consideration factors that differed between studies. Hence, the model needs adaptation to accurately describe the existing data, in addition to empirical evaluation of its predictions.

### Conflict of interest statement

The author declares that the research was conducted in the absence of any commercial or financial relationships that could be construed as a potential conflict of interest.

## Footnotes

^1^A related phenomenon is the mismatch negativity (MMN; Näätänen et al., 1978). The MMN is a relative negativity to expectancy-violating deviants compared to expectancy-matching standards, which is typically measured in oddball tasks. The MMN can be explained by sensory adaptation (for a review see May and Tiitinen, 2010) or by an involvement of top–down expectations or their violation (e.g., Schröger and Wolff, 1996; see also Todorovic et al., 2011; Todorovic and de Lange, 2012). In classical MMN-studies, the MMN is mostly related to an enhanced negativity to deviants, hence measuring expectancy-violation. However, the early negativity to standard stimuli has been shown to decrease with an increasing number of repetitions (and hence increasing predictability) of the standard (Haenschel et al., 2005; see also Todorovic et al., 2011; Todorovic and de Lange, 2012). This suggests that the MMN is partly due to a reduction of negativity as a function of the predictability of the standards (see also Baldeweg, 2007 for discussion).

^2^Note that some related studies cannot be classified unequivocally in this taxonomy: Correa and Nobre (2008) and Rohenkohl and Nobre (2011) share features of both probabilistic and rhythmic cuing.

^3^Note that the rhythmic cuing study by Rimmele et al. (2011), cannot be classified with respect to this distinction, since conditional probability is high for the regular condition but varies for the irregular condition.

^4^At first glance, the findings of the recent study by Sanabria and Correa (2013) are inconsistent with those of Lange (2010): These authors found reduced negativities to rhythmically cued targets—although stimulus sequences did not reliably predict sound onset. However, Sanabria and Correa (2013) compared the processing of a valid and an invalid condition. Hence, the effect may either reflect a reduced negativity to validly cued targets or an enhanced negativity to invalidly cued targets (akin to a mismatch response, e.g., Schröger, 1998).
